# Supplementary material for: A whole-animal phenotypic drug screen identifies suppressors of atherogenic lipoproteins
Source: eLife. 2026 Aug 27;14:RP105314. doi: 10.7554/eLife.105314 (PMC13521566; doi:10.7554/eLife.105314)
Supplement: Supplementary file 2 [file elife-105314-code1.zip › JHDL_primary_result/Diphenylboric acid/Diphenylboric acid_JHDL_RARE_020_065_2020_02_07_12_20.pdf]

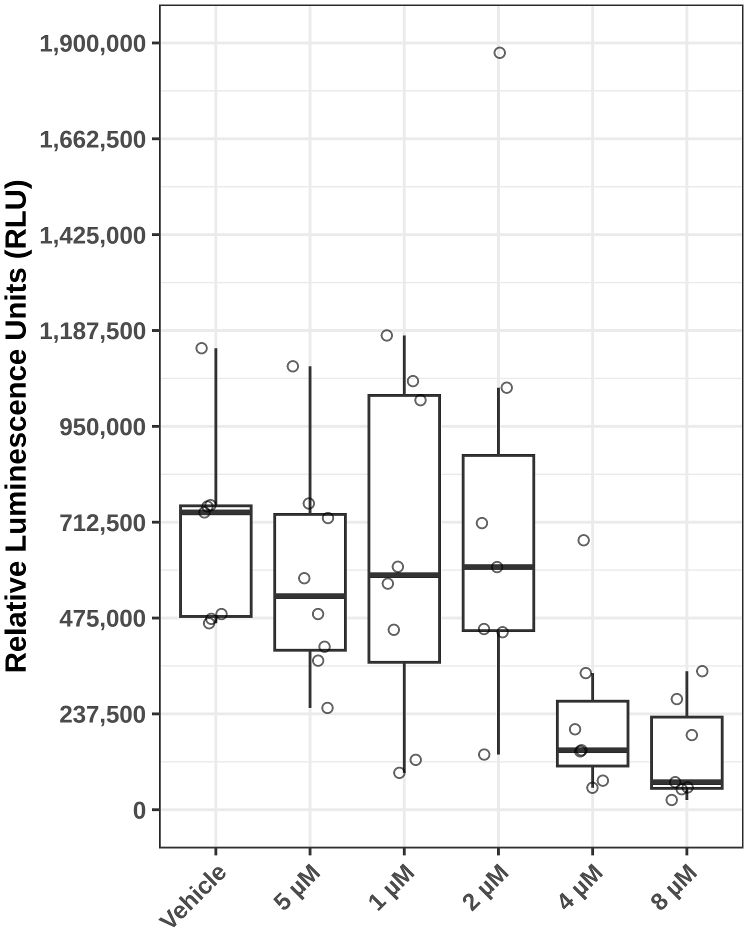

Relative Luminescence Units (RLU)
1,900,000
1,662,500
1,425,000
1,187,500
950,000
712,500
475,000
237,500
0
Vehicle
5 μM
1 μM
2 μM
4 μM
8 μM
